# Neutrophil swarms containing myeloid-derived suppressor cells are crucial for limiting oral mucosal infection by C. albicans

**DOI:** 10.21203/rs.3.rs-3346012/v1

**Published:** 2023-10-09

**Authors:** Mira Edgerton, Isolde Rojas, Rohitashw Kumar, Rui Li, Ornella Salvatori, Scott Abrams, Daniel Irimia

**Affiliations:** SUNY Buffalo; SUNY Buffalo; SUNY Buffalo; SUNY Buffalo; SUNY Buffalo; Roswell Park Comprehensive Cancer Center; Massachusetts General Hospital

## Abstract

Oral mucosal colonization by *C. albicans* (Ca) is benign in healthy people but progresses to deeper infection known as oropharyngeal candidiasis (OPC) that may become disseminated when combined with immunosuppression. Cortisone-induced immunosuppression is a well-known risk factor for OPC, however the mechanism by which it permits infection is poorly understood. Neutrophils are the primary early sentinels preventing invasive fungal growth, and here we identify that *in vivo* neutrophil functional complexes known as swarms are crucial for preventing Ca invasion which are disrupted by cortisone. Neutrophil swarm function required leukotriene B4 receptor 1 (BLT1) expression, and swarms were further characterized by peripheral association of polymorphonuclear myeloid-derived suppressor cells (PMN-MDSCs) showing that OPC recruits PMN-MDSCs to this site of infection. Furthermore, PMN-MDSCs associated with Ca hyphae had no direct antifungal effect but showed prolonged survival times and increased autophagy. Thus *in vivo* neutrophil swarms are complex structures with spatially associated PMN-MDSCs that likely contribute immunoregulatory functions to resolve OPC. These swarm structures have an important function in preventing deep invasion by Ca within the oral mucosa and represent a mechanism for increased disease severity under immune deficient clinical settings.

## INTRODUCTION

The oral epithelium provides both mechanical and immunological barriers against the opportunistic fungus *Candida albicans* that causes oropharyngeal candidiasis (OPC), also known as thrush. Individuals with immune deficiencies or impaired immunity due to corticosteroid therapy ^5^ are especially susceptible to both OPC and deeper fungal infection. However, surprisingly little is known about the mechanisms by which corticosteroids such as cortisone initiate OPC, despite their widespread clinical use such as with oral inhalers.

Neutrophil recruitment is an important first line to defense in host response to OPC ^6–8^. Cortisone-treated mice infected with *C. albicans* form white tongue plaques consisting of an adherent biofilm that disrupts the tongue filiform papillae and hyphae that penetrate deep into the corneum stratum (CS) ^9, 10^. Although fungal invasion recruits massive numbers of neutrophils that infiltrate throughout the epithelium and connective tissue (CT), they are unable to resolve *C. albicans* infection in cortisone-induced immune suppressed animals ^9^. Similarly, mice deficient in IL-17 receptor signaling show persistent fungal infection despite extensive neutrophilic infiltration ^6, 10, 11^. Thus, the function of neutrophils in resolving OPC has been questioned in the context of immunodeficiencies.

Epithelial infection by *C. albicans* causes a temporal release of an array of cytokines and chemokines that recruit and activate neutrophils. IL-1 signaling induces expression of granulopoietic cytokines such as G-CSF in the connective tissue, leading to production and release of neutrophils from the bone marrow into the circulation ^7^. Epithelial cells also produce neutrophil chemokines, including KC/CXCL1 and MIP-2/CXCL2, that induce infiltration of polymorphonuclear neutrophils into the oral mucosa ^6–8^. Glucocorticoids are known to inhibit neutrophil recruitment by affecting several steps in the extravasation cascade ^16^. Furthermore, chronic exposure to glucocorticoids can shift the balance of the innate immune response resulting in dysregulated expression of chemokines and pro-inflammatory cytokines ^17^. Thus, cortisone-induced OPC might be a consequence of delayed neutrophil recruitment, although the large numbers of neutrophils located within *C. albicans* infected epithelium suggests other functional defects.

Recently a novel function of neutrophils has been identified in which neutrophils switch from simple chemotaxis towards the site of bacterial, fungal or parasitic infection to a swarm-like migration pattern known as neutrophil swarming ^12^. Neutrophil swarming is characterized by highly coordinated chemotaxis and accelerated neutrophil accumulation to form clusters around the invading microorganism, thus insulating the infected site from surrounding healthy tissue ^13^. Neutrophil swarms can be transient (small diameter of < 150 neutrophils,) or permanent (large diameter of > 300 neutrophils) in nature ^12, 14^. Initiation of swarm formation is controlled by leukotriene B4 and interaction with its receptor BLT1 (also known as LTB4R1) ^15^. Other mediators contributing to swarm formation in mice include CXCR2 ligands, such as CXCL2 (MIP-2). Hopke et al. (2020) showed that neutrophil swarming is a response to *C. albicans* infection *in vitro*, involving LTB4/BLT1 and G-CSF signaling for swarm formation, and MPO for *C. albicans* killing. However, there is limited *in vivo* evidence for the function of neutrophil swarms beyond those reported by intravital microscopy in bacteria infected murine skin and liver ^12^.

Although neutrophils were considered a homogeneous population when compared to macrophages and lymphocytes, several studies have now demonstrated intrinsic functional heterogeneity in the circulating neutrophil pool (reviewed in ^18^). A specific subset of immunomodulatory cells expressing high levels of Arginase 1 (Arg1) ^19^ are known as myeloid-derived suppressor cells (MDSCs) and have been well described when infiltrating tumors and in hyperproliferative skin disorders ^20^. In mice, two major MDSC subsets are identified by their differential expression of the Ly6G and Ly6C surface antigens and by their immunosuppressive activities, in part mediated by the expression of the enzyme Arg1. Monocytic (M) MDSCs are defined as being CD11b^+^Ly6G^neg^Ly6C^high^, while granulocytic or polymorphonuclear (PMN) MDSCs are CD11b^+^Ly6G^+^Ly6C^low 21^. Although they express similar surface markers as neutrophils, PMN-MDSCs can be differentiated from neutrophils by their ability to suppress T cell proliferation ^22^. One recent study showed that systemic *C. albicans* infection induces mainly the PMN-MDSC subset where they have a protective role by limiting host hyperinflammatory responses ^23^. Furthermore, adoptive transfer of MDSCs conferred protection against systemic fungal dissemination and increased murine survival ^23^. However, it is not known whether MDSCs have any biological role in localized fungal mucosal infections such as OPC.

In this work we show that cortisone immunosuppression not only delayed cytokine / chemokine expression and recruitment of neutrophils in response to *C. albicans* oral infection but disrupted *in vivo* BLT1 signaling and neutrophil swarming. Furthermore, we found that neutrophils swarms contained spatially localized PMN-MDSCs that associated with *C. albicans* hyphae and are components of *in vivo* swarms. Our findings provide a mechanistic explanation for how cortisone is a significant risk factor for OPC and supply *in vivo* evidence that neutrophil swarming is a crucial component for resolving *C. albicans* oral infection.

## RESULTS

### Cortisone both delayed inflammatory cell recruitment and altered their ability to control infection.

**Figure 1:**
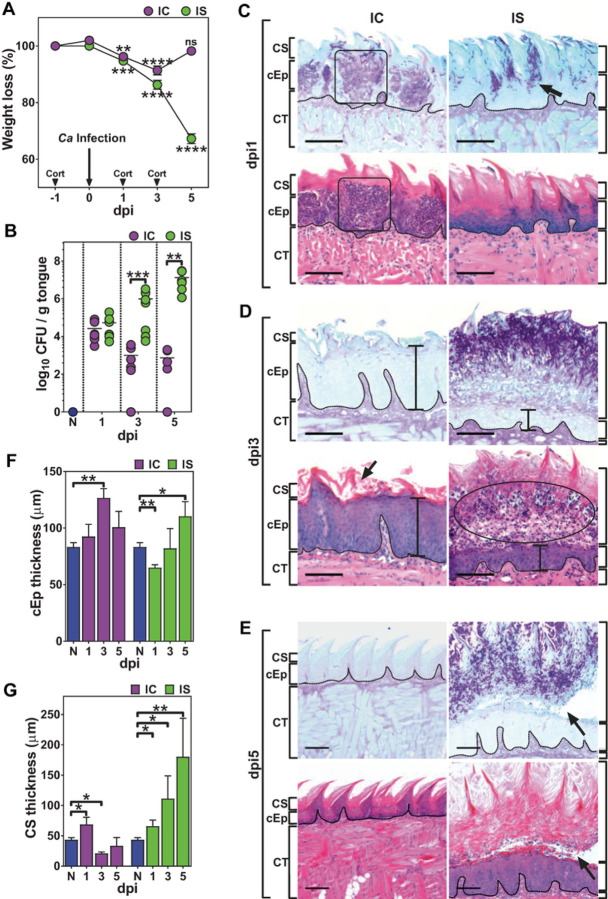
Immunosuppression (IS) impairs inflammatory cell recruitment and alters epithelial morphology. Cortisone-treated mice (IS) and immunocompetent (IC) mice were infected sublingually with 10^7^ CFU of *C. albicans* (Ca) and tongues were collected from 1 to 5 days post-infection (dpi) (5–7 mice/ group). A) Percentage of weight loss after Ca infection as compared to pre-infection weight. B) Number of CFU/ g tongue tissue in IC and IS mice shows rapid clearance of Ca in IC mice and a persistence of infection in IS mice. C-E) Representative co-localized tongue sections from IC and IS mice after Ca infection in IC and IS mice stained for PAS and H&E (n=3 mice/group). C) IC mice show early and robust intraepithelial recruitment of inflammatory cells to the areas of candida invasion as compared to IS mice (square) (dpi1). D) Vertical bars show differences in cellular epithelial thickness (cEp) between IC and IS mice at dpi3. E) Arrow indicates detachment of corneum stratum from underlying cEp in IS mice at dpi5. F) Changes in cEp thickness (mm) and (G) and corneum stratum thickness as compared to naive (N) mice from dpi1-3. ** p< 0.01; *** p< 0.001; **** p<0.0001 (Mann-Whitney or 2-tailed t-test). Scale bar=100 mm

To determine whether cortisone affects only neutrophil recruitment, we compared *C. albicans* oral infection between immunocompetent (IC) and cortisone immunosuppressed (IS) mice histologically on days post-infection (dpi) 1,3 and 5. IC and IS animals had similar total body weight loss (Fig. 1A) and infection levels as measured by Ca CFU (Fig. 1B). But at dpi1, we observed striking differences in inflammatory cell recruitment (Fig. 1C). IC animals had extensive inflammatory cell infiltration primarily in the cellular epithelium (cEp), but also extending into the corneum stratum (CS) and connective tissue (CT). These inflammatory cells (consisting of multi-lobulated nuclear morphology characteristic of neutrophils) were well organized and tightly clustered around and beneath invading fungal cells as in swarms (black boxes, Fig. 1C). In contrast, IS animals had no evidence of inflammatory cell recruitment despite having fungal invasion deep into the cEp that nearly reached the basement membrane (arrow).

By dpi 3, IC tongues showed a one log-fold reduction in fungal burden and fungal cells were mainly isolated to the uppermost CS layers (Fig. 1D, arrow). Histological H&E assessment of inflammatory cell infiltration showed that it was largely absent in the cEp and confined to the CT (Fig. 1D). Interestingly, resolution of infection in IC animals was accompanied by increased hyperplastic thickening of the cEp (Fig. 1D black bars and Fig. 1F) as well as thickening of the CS (Fig. 1G). IS animals at dpi3 had significantly more weight loss than IC animals (Fig. 1A) along with a one log-fold increase in Ca CFU compared to dpi1 and profuse recruitment of inflammatory cells to the cEp (Fig. 1D, circle). However, there was a remarkable lack of organization in inflammatory cells at the infection site, and these cells were widely dispersed instead of forming clusters as found in IC animals. The thickened cEp response was not found in IS animals, however the CS layer was significantly increased (Fig. 1G) as a result of the mass of fungal hyphae localized in this area (Fig. 1D).

At dpi5, IC animals weight returned to pre-infection levels and tongue tissues had histologically normal architecture and cEp thickness (Fig. 1E). However, the epithelium retained 10^3^ CFU/g tissue (Fig. 1B) with little visible inflammatory cell infiltration (Fig. 1E). In contrast, IS animals had significant morbidity along with continued loss of body weight that required sacrifice (Fig. 1A). Ca infection levels in tongues climbed to 10^7^ CFU/g (Fig. 1B) that was mainly found within the CS (Fig. 1E). Inflammatory cells were prolific and widely distributed without the organization of swarms observed in IC tissues. These large numbers of inflammatory cells significantly increased the thickness of the CS that was three-fold more than that found in IC animals at any time point (Fig. 1G). The cEp in IS animals was only slightly thickened, but we observed several areas in which CS desquamation (containing large masses of fungi) from the underlying cEp occurred (Fig. 1E, arrow), resulting in exfoliation and further seeding of Ca into the oral environment. Thus, cortisone caused significant delays in inflammatory cell recruitment as well as loss of organized structure of inflammatory cells recruited by dpi5, resulting in ineffective reduction of Ca infection levels.

### OralC. albicansinfection leads to recruitment of neutrophils but not macrophages.

**Figure 2:**
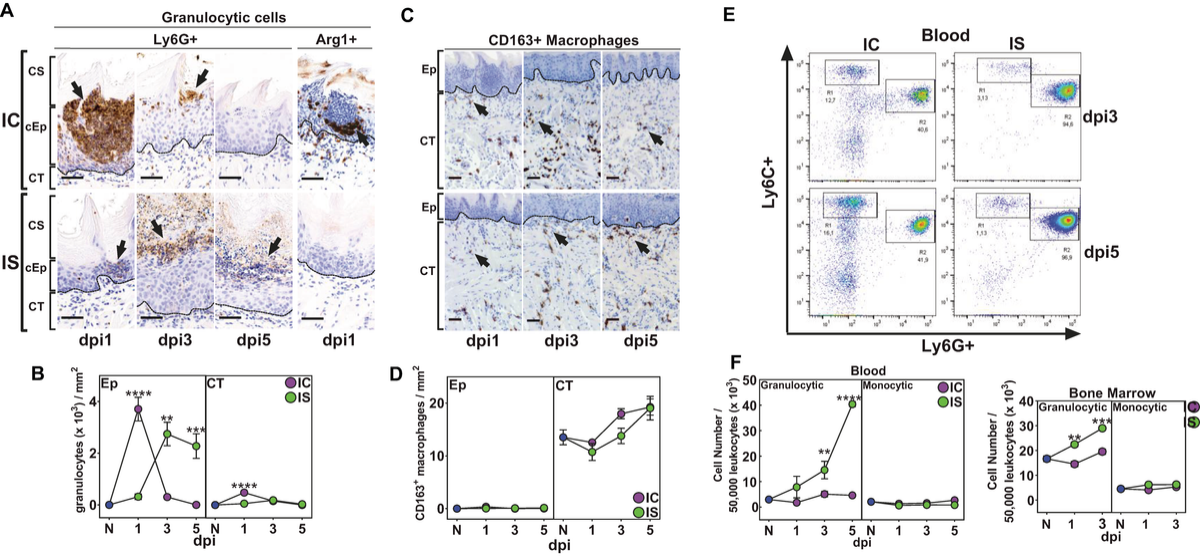
Oral fungal infection leads to increased recruitment of neutrophils but not macrophages. Representative slides showing recruitment of Ly6G+ granulocytes and a subset of Ly6G+ Arginase (Arg) 1+ granulocytes into the tongue epithelium (Ep) of IC and IS mice. B) Changes in recruitment of Ly6G+MPO+ granulocytes / mm^2^ (x10^3^) from IC and IS mice as compared to naive (N) at dpi1-5 (n=3 / group). C) Representative slides showing recruitment of CD163+ macrophages into the connective tissue (CT) and not the Ep of both IC and IS mice. D) Changes in recruitment of CD163+ macrophages / mm^2^ in IC and IS mice as compared to N at dpi1-5. E) Representative FACS plots of CD45+CD11b+Ly6G^high^ granulocytic cells and CD45+CD11b+Ly6G^lo^ monocytic cells in blood collected from IC and IS mice at dpi3-5 showing a significant expansion of granulocytic cells and not monocytic cells. F) Changes in monocytic and granulocytic cells in blood (dpi1-5) and bone marrow (BM) (dpi1-3) collected from IC and IS mice as compared to N (n= 3–7 mice/group, except for dpi 5 where n=2 mice/group). * p<0.05; ** p< 0.01; *** p< 0.001; **** p<0.0001 (Mann-Whitney or 2-tailed t-test). Scale bar=50 mm

To quantitate inflammatory cell recruitment to the site of oral fungal infection, epithelial and CT compartments were measured histologically for Ly6G + neutrophils and CD163 + macrophages. Inflammatory cells localized in the Ep following fungal infection in both IC and IS tongues were predominantly Ly6G + confirming their identity as neutrophils (Fig. 2A). We also observed these cells to be highly organized in Ep tissues on dpi1 in IC animals in contrast to the diffuse infiltrate in IS Ep tissues (Fig. 2A). Furthermore, a small subset of granulocytes adjacent to and within the neutrophil swarms were Ly6G + Arg1+, also consistent with the accumulation of granulocytic or polymorphonuclear (PMN) MDSCs (Fig. 2A). Surprisingly, macrophages were localized exclusively to CT and did not enter the Ep in either IC or IS animals (Fig. 2C).

Quantification of neutrophils showed that these cells were recruited primarily to the Ep at dpi1 in IC animals, as well as a small yet significant increase in neutrophil infiltration in the CT at dpi1 (Fig. 2B). As we observed histologically, IS animals had delayed recruitment of neutrophils, but the number of cells recruited at dpi3 was like that of IC animals at dpi1 and persisted in Ep through dpi5. In contrast, macrophages were absent from the Ep and localized in low numbers within the CT compartment in both IC and IS tongues at all time points (Fig. 2C). However, the numbers of macrophages cells in IC and IS animals were not significantly different than naive mice showing that oral fungal infection did not elicit further recruitment of macrophages to oral CT (Fig. 2D).

To determine systemic inflammatory cell production in response to Ca oral infection, we examined numbers of granulocytic cells (CD11b^+^Ly6G^+^Ly6C^low^) which may include neutrophils and PMN-MDSCs, and monocytic cells (CD11b^+^Ly6G^−^Ly6C^high^) which may include M-MDSCs and macrophage precursors. Flow cytometry analyses of bone marrow and peripheral blood showed that systemic production of inflammatory cells was skewed towards granulocytic cells in response to oral Ca infection (Fig. 2E). Granulocytes were significantly elevated in both bone marrow (dpi1 and dpi3) and blood (dpi3) in IS animals compared to IC animals (Fig. 2F). By dpi5, IS animals had a four-fold higher expansion (p < 0.001) of peripheral granulocytic cells compared with IC animals that had returned to basal levels (Fig. 2F). Thus, cortisone increased granulocyte expansion in response to oral Ca infection in both the bone marrow and peripheral blood. Surprisingly, monocyte levels were unchanged in the bone marrow and peripheral blood in IC and IS animals, showing that oral Ca infection had little impact on monocyte/macrophage production and recruitment. Thus, based upon frequencies, monocytic cell (macrophage or dendritic cell progeny) expansion and recruitment does not appear to have a role in the response to oral fungal infection, with the predominant response being granulocytic cell accumulation including neutrophils and possibly PMN-MDSCs.

### Expansion of PMN-MDSCs in response to oral candidiasis was confirmed by the immunosuppressive activity on CD4 + T cells by Ly6G + Arg1 + cells.

**Figure 3:**
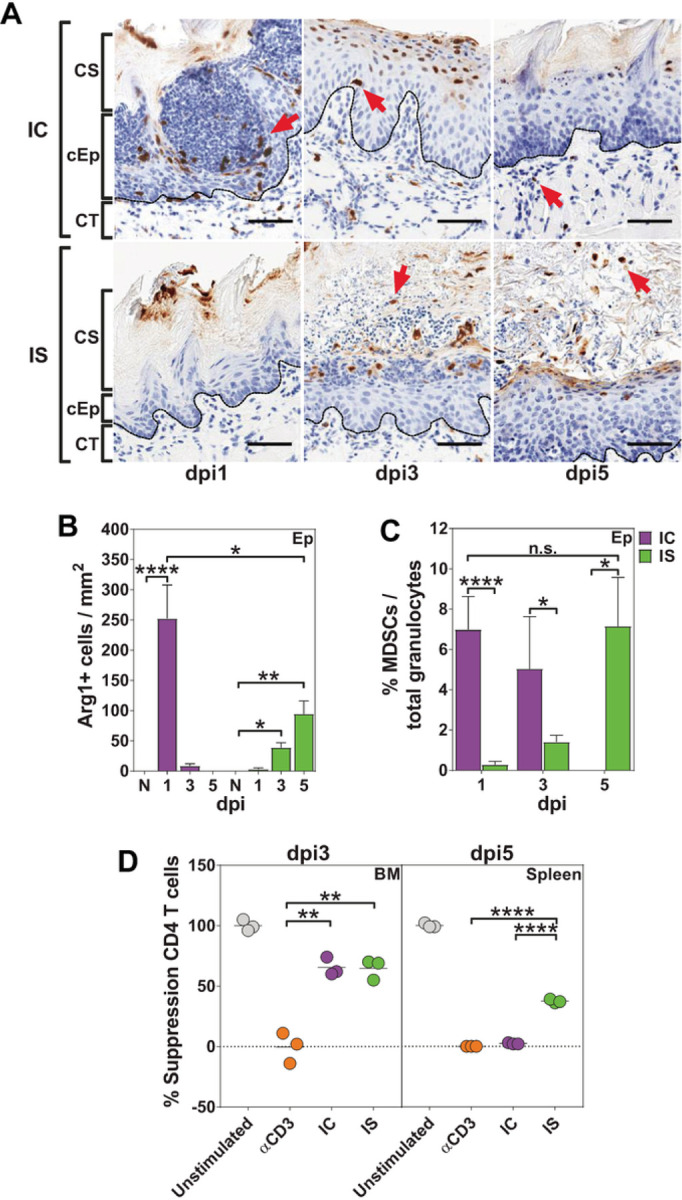
Ly6G+ Arg1+ cells show immunosuppressive activity on CD4+ T cells confirming the expansion of PMN-MDSCs in response to oral candidiasis. A) Representative slides showing epithelial infiltration of Ly6G+ Arg+ cells in IC and IS mice on dpi 1–5. B) Ly6G+ Arg+ granulocytes/ mm^2^ show peak recruitment on dpi1 for IC mice and dpi3-5 for IS mice (3–4 mice / group). C) Percentages of Ly6G+Arg+ granulocytes to total Ly6G+ granulocytes were higher on dpi 1 for IC and dpi 5 for IS mice. D) CD11b+ Ly6G+ enriched single cells suspensions from BM and spleen of IC and IS mice showed inhibition of spleen-derived CD4+ T cell proliferation on dpi3-5 (graphs are representative of two independent experiments). * p< 0.05; ** p< 0.01; *** p< 0.001; **** p<0.0001 (t-test). Scale bar=50 mm

We found that recruitment and localization of Ly6G + Arg1 + cells to infected tongue Ep tissues was affected by cortisone. In IC animals, Ly6G + Arg1 + cells were localized to the periphery of neutrophil swarms on dpi1 (Fig. 3A red arrows), then were reduced in the Ep at dpi3 and by dpi5 all were confined to the CT compartment. In contrast IS animals had no epithelial recruitment of Ly6G + Arg1 + until dpi3 and those were randomly scattered throughout disorganized neutrophilic areas. Total numbers of Ly6G + Arg1 + cells recruited to Ep were significantly higher in IC animals at dpi1 than at any time point in IS animals (Fig. 3B), although the proportion of Ly6G + Arg1 + to total granulocytes was equivalent between IC dpi1 and IS dpi5 (Fig. 3C). Thus, IC animals recruited higher total numbers of Ly6G + Arg1 + cells to the Ep in response to Ca infection while IS animals had reduced and delayed Ly6G + Arg1 + recruitment.

Next, we examined Ly6G + cells from the bone marrow and spleen as proxy sites for the myeloid response in IC and IS mice for their ability to inhibit T-cell proliferation to confirm their identity as PMN-MDSCs (Fig. 3D). Bone marrow-derived CD11b^+^Ly6G^+^Ly6C^low^ cells collected from both IC and IS mice dpi3 caused 60% suppression in CD4 + T cell proliferation, confirming the functional expansion of PMN-MDSCs in the granulocyte pool (Fig. 3D). On dpi5, the immunosuppressive effects of CD11b^+^Ly6G^+^Ly6C^low^ cells, as measured from the spleen, only persisted in IS mice, suggesting a protracted expansion of PMN-MDSCs in IS mice as a result of continued Ca infection. Thus, PMN-MDSCs are recruited to the tongue as a part of the systemic granulocytic response to Ca infection.

### Neutrophil swarming and BLT1 expression are reduced by cortisone.

**Figure 4:**
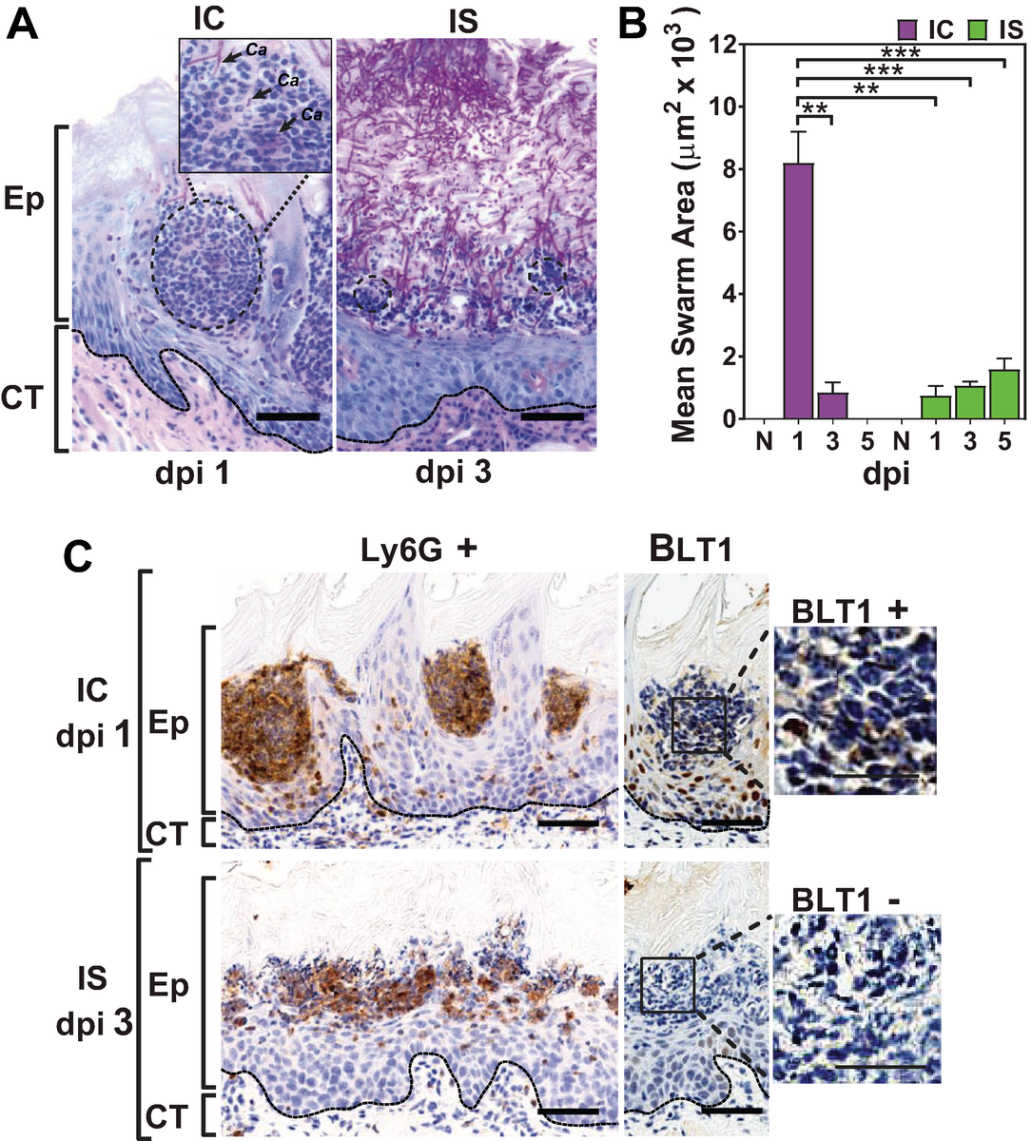
Neutrophil swarming and BLT1 expression are reduced by immunosuppression. A) PAS staining of tongue sections showing the association between neutrophil swarms (circles) and Ca. B) Measurements of mean swarm area (mm^2^ × 10^3^) showed significant swarm area reduction in IS mice on dpi1-5 as compared to the swarm area of IC mice on dpi1. B) Representative tissue sections showing formation of multiple intraepithelial Ly6G+ BLT1+ neutrophil swarms in IC mice (upper panel), whereas IS mice show minimal swarm formation and absence of BLT1 expression. ** p< 0.01; *** p< 0.001; **** p<0.0001 (t-test). Scale bar=50 mm (25 mm for enlarged BLT-1 insert) (n=3 mice / group per time point).

If swarming is affected by cortisone, we expected to see disrupted swarm morphology and BLT1 expression in IS animals. Indeed, IC animals produced neutrophil swarms that were readily visualized within the infected Ep (Fig. 4A, left panel, circle) in which neutrophils tightly surrounded the invading Ca hyphae and yeast (enlarged view), while IS animals had a diffuse neutrophilic infiltration that sometimes produced very small swarm-like nodules (transient swarms) (Fig. 4A, right panel, small circles). We next measured mean swarm areas from multiple tongue sections from IC and IS animals. Quantification of these areas showed that IC swarms were 8-fold larger at dpi1 than IS animals produced at any time point during Ca infection (Fig. 4B). A key factor required for swarming is localized expression of the chemoattractant leukotriene B4 that is recognized by the high affinity leukocyte receptor BLT1 ^15^. Therefore, we examined expression of BLT1 in neutrophils recruited to the infection site. Neutrophils were visualized by Ly6G staining in Ca infected tissues in IC and IS animals, then probed for BLT1 expression. We found that neutrophils forming organized swarms in IC animals also highly expressed BLT1 (Fig. 4C upper panel), while none of the neutrophils in IS animals were found to express BLT1 at levels detectable by immunohistochemistry (Fig. 4C lower panel). This absence of BLT1 expression was consistent in IS animals from dpi3 through dpi5. Thus, one mechanism by which cortisone disrupts neutrophil swarm formation is by reduction of leukotriene B4 / BLT1 signaling.

### Cortisone induced immunosuppression delays expression of proinflammatory cytokines and chemokines in response to fungal infection.

**Figure 5:**
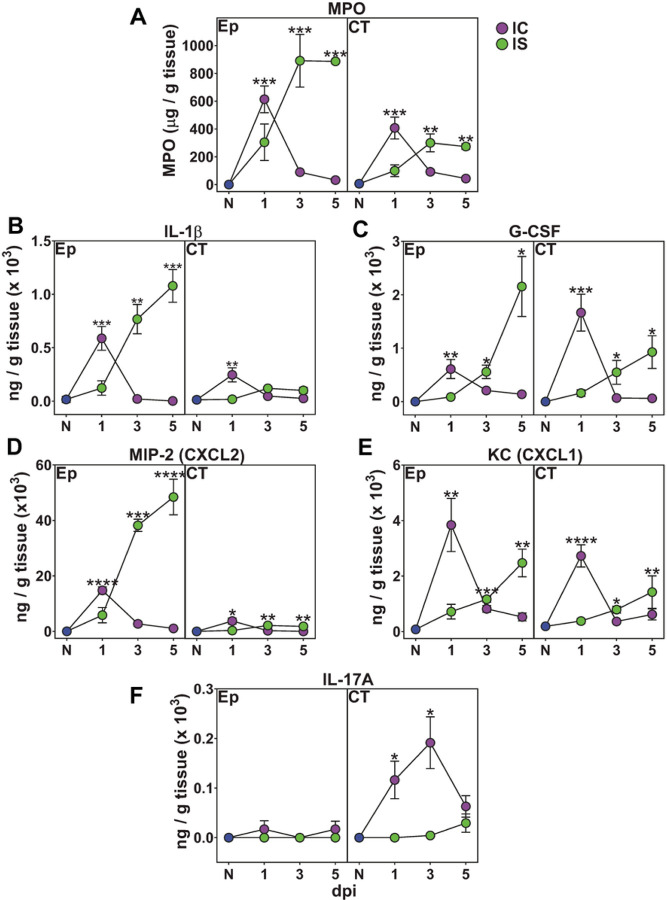
Cortisone-induced immunosuppression delays expression of proinflammatory cytokines and chemokines in response to fungal infection. Freshly extracted tongue tissues were dissected for separate protein isolation in epithelium (Ep) and connective tissue (CT) (n=5 mice/group per time point). A) Kinetics of MPO (ELISA) used as a surrogate marker of neutrophil and PMN-MDSC recruitment in IC and IS mice as compared to naive (N) mice. B-D) Kinetics of IL-1b (B), G-CSF (C), MIP-2 (D), and KC (E) (measured by Bio-Plex assay) in IC and IS mice as compared to N mice followed a similar pattern as MPO in dpi1-5 at Ep and CT. F) IL-17 was increased only in CT of IC mice, and not in IS mice, as compared to naïve (N) mice (dpi1-3). * p< 0.05; ** p< 0.01; *** p< 0.001; **** p<0.0001 as compared to naïve (N) mice (ANOVA and Tukey’s multiple comparisons)

We next asked whether cortisone altered oral tissue specific expression of key inflammatory cell recruitment cytokines that may skew neutrophil recruitment or swarming. We examined Ep and CT expression levels of the cytokines IL-1β and G-CSF, known to expand and activate neutrophils during OPC, and the chemokines KC/CXCL1 and MIP-2/CXCL2, which recruit neutrophils in response to oral candidiasis in tongue tissue ^6–8^ (Fig. 5B–E). We also examined tissue levels of IL-17A that may have a role in neutrophil recruitment (Fig. 5F). We examined Ep and CT myeloperoxidase (MPO) as a marker of neutrophil recruitment and found maximal levels at dpi1 in the Ep of IC animals that were delayed until dpi3 in IS animals (Fig. 5A). Among the neutrophil recruitment/activation chemokines and cytokines examined, all four (IL-1β, G-CSF, KC/CXCL1 and MIP-2/CXCL2) demonstrated temporal expression levels that very closely paralleled neutrophil recruitment in both IC and IS animals (Fig. 5B–E), suggesting that defective expression among these cytokines is unlikely to be the reason for loss of swarming behavior. However, cortisone induced immunosuppression delayed the expression of these cytokines and chemokines at dpi1 accounting for delayed neutrophil recruitment. We found that MIP-2/CXCL2 and IL-1β expression to be mainly localized to the Ep, while G-CSF and KC/CXCL1 were expressed in both Ep and CT. MIP-2/CXCL2 expression was the most upregulated chemokine tested, being 3- to 10-fold higher than others measured at dpi1 in IC and at dpi 3–5 in IS animals. In contrast, while endogenous IL-17A production was restricted to the CT (not detectable in Ep) in IC tissues, its expression was completely suppressed by cortisone at dpi1 and dpi3 (Fig. 5F), suggesting that CT-localized innate immune cells expressing IL-17 play some biological role in the process of neutrophil recruitment.

### Treatment with anti-Ly6G antibody caused disruption of neutrophil swarming and increased tissue invasion of Ca.

**Figure 6:**
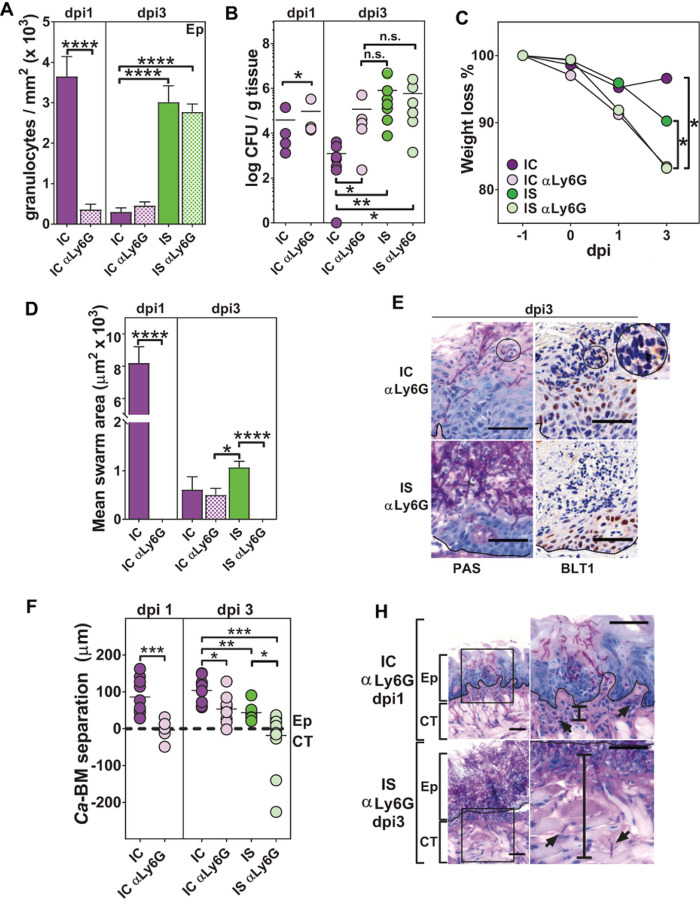
Treatment with anti-Ly6G caused disruption of neutrophil swarming and increased Ca invasion into the connective tissue. Neutrophils were depleted by i.p. injection of anti-Ly6G antibody on dpi-1 and anti-GCF antibody on dpi-1 through 2 (n=3–6 mice/ group per time point). Depletion was confirmed by flow cytometry. A) Ly6G+ cell depletion resulted in a significant reduction in total neutrophils in IC mice on dpi1-3, whereas IS mice showed increased neutrophil recruitment on dpi3 regardless of depletion. B) Number of CFU / g tongue tissue in anti-Ly6G+ treated IC and IS mice showing delayed clearance of Ca and a persistence of infection on dpi 3 comparable to isotype-treated IS mice. C) Anti-Ly6G+ treatment resulted in significant weight loss in IC and IS mice as compared to isotype-treated IC and IS mice by dpi 3. D) Ly6G+ cell-depletion in IC mice resulted in significant reduction in swarm formation on dpi1, with small swarm formation on dpi3, whereas depletion caused complete disruption of swarm formation in IS mice on dpi3. E) Representative co-localized sections for PAS and BLT1 staining, showing small BLT-1+ swarm formation (circle) in IC anti-Ly6G+ cell-depleted mice on dpi 3, however, IS anti-Ly6G+ mice showed absence of swarm formation and BLT1 expression. F) The distance (mm) was measured between Ca and the basement membrane (BM) in PAS-stained tissue sections from IC and IS mice showing that anti-Ly6G+ treatment resulted in Ca invasion of connective tissue on dpi1-3. H) Representative PAS-stained slides showing CT invasion of Ca hyphae (distance indicated by vertical bar) in anti-Ly6G+ treated IC and IS mice. * p< 0.05; ** p< 0.01; *** p< 0.001; **** p<0.0001 (t-test, ANOVA and Tukey’s multiple comparisons). Scale bar=50 mm (25 mm for enlarged images).

To determine whether cortisone had additional effects beyond delaying recruitment of neutrophils, we treated animals with anti (α)-Ly6G antibody to specifically reduce early neutrophil recruitment then measured effects on swarming and animal morbidity (CFU, weight loss and tissue invasion). We expected that if cortisone solely functioned to reduce swarm formation, then α-Ly6G treatment in IC animals (IC α-Ly6G) would have equivalent infection outcomes as IS or ISα-Ly6G control animals. α-Ly6G treatment reduced total tissue granulocytic recruitment in IC animals at dpi1 (Fig. 6A). However, by dpi3 total granulocyte numbers in tissues did not differ between groups with or without α-Ly6G treatment (and IS compared to IS α-Ly6G). Ca infection levels (CFUs) were significantly increased in IC α-Ly6G compared to IC animals at both dpi1 and dpi3 (Fig. 6B), however IC α-Ly6G, IS, and IS α-Ly6G groups all had equally elevated CFUs despite different total numbers of recruited granulocytes. Strikingly, animal morbidity as measured by weight loss was most severe in α-Ly6G treated animals (IC α-Ly6G and IS α-Ly6G animals lost significantly more weight than IS animals) requiring early sacrifice by dpi3 (Fig. 6C). However, total numbers of infecting Ca tongue CFUs and total granulocyte numbers did not account for this increased weight loss.

We next measured neutrophil swarming behavior among these groups expecting that α-Ly6G treatment would reduce numbers of neutrophils and have impaired swarming (Fig. 6D). As expected, IC α-Ly6G mice at dpi1 with reduced granulocyte numbers showed nearly complete loss of swarms. At dpi3, α-Ly6G treated animals (IC α-Ly6G and IS α-Ly6G) had significantly smaller swarm areas than IS mice, that was consistent with weight loss in each group. IC α-Ly6G tissues still contained some small mini-swarms with detectable BLT1 staining (Fig. 6E, circles); however, IS α-Ly6G animals showed in a complete loss of swarms (despite having abundant numbers of neutrophils) and undetectable BLT1 expression. Thus, cortisone caused more profound loss of BLT1 expression than α-Ly6G treatment although both have the same target.

To assess the impact of impaired swarm formation, we examined severity of infection by assessing the depth of Ca invasion relative to the basement membrane (Ca-BM separation, BM shown in dotted lines Fig. 6F), since invasion into the CT is permissive for dissemination. There was a significant increase in Ca invasion depth in IC α-Ly6G animals at dpi 1, with many animals showing CT invasion (Fig. 6H, black bars show CT invasion depth). At dpi3, both IC α-Ly6G and IS animals had significantly increased invasion compared to IC (Fig. 6F), although increased cEp thickening in the IC group may have mitigated apparent invasion depth. Strikingly, Ca invaded up to 200 µm into CT in IS α-Ly6G animals (Fig. 6H, lower panel), despite having equal numbers of neutrophils to IS animals in which the invasion depth was mainly restricted to the Ep. Thus, animals that exhibited the greatest CT invasion depth were those with the greatest loss of swarming behavior (IC α-Ly6G dpi1 and IS α-Ly6G dpi3), that could not be accounted for by absolute differences in neutrophil numbers or CFUs.

### PMN-MDSCs are differentially localized within swarms in IC and IS mice, although both express COX-2.

**Figure 7:**
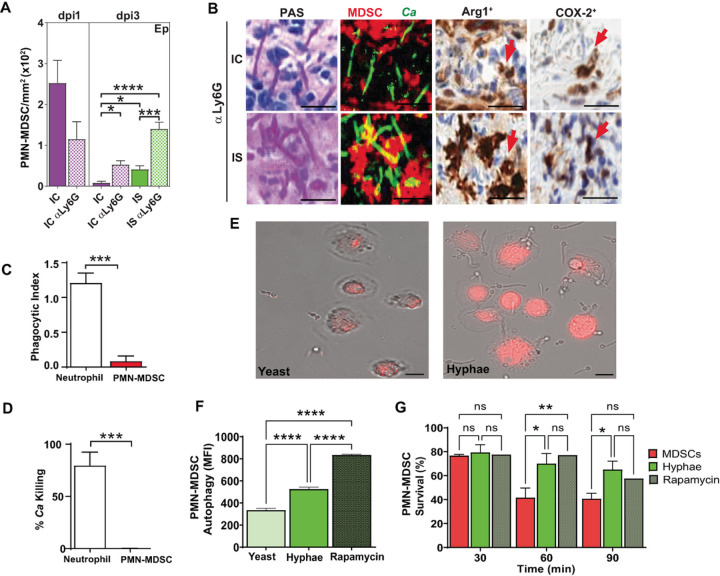
Anti-Ly6G+ treatment did not change recruitment of PMN-MDSCs in IC and IS mice, and human PMN-MDSCs showed absence of Ca killing and phagocytic uptake of Ca. A) Number of PMN-MDSCs / mm^2^ was increased by anti-Ly6G+ treatment in IC and IS mice on dpi3 (n=3–5 mice / group per time point). B) Co-localized PAS, Arg 1+ and COX-2 stained tongue tissue sections show co-localization with Ca of Arg+ granulocytes by image deconvolution, along with expression of COX-2 in IC and IS anti-Ly6G+ treated mice. C) Human PMN-MDSCs showed significantly lower (10-fold) phagocytic index of Ca cells, compared with human neutrophils. D) Fungicidal activity was not detected in human PMN-MDSCs cells. E) *In vitro* Ca yeast cells showed very little contact with PMN-MDSCs, while Ca hyphae were closely associated with the surfaces of PMN-MDSCs. F) Autophagy levels of human PMN-MDSCs (MFI) incubated with Ca hyphae cells reached 70% that of rapamycin, and was significantly higher than that incubated with Ca yeast cells. G) PMN-MDSCs survival was increased by 50% after 60 min exposure to Ca hyphae. * p< 0.05; ** p< 0.01; *** p< 0.001; **** p<0.0001 (t-test, ANOVA and Tukey’s multiple comparisons). Scale bar=25 mm

Since we observed PMN-MDSCs to be components of swarms, we questioned whether PMN-MDSCs recruitment was also affected by α-Ly6G treatment that might account for differences in Ca tissue invasion. Numbers of PMN-MDSCs were not significantly different in IC α-Ly6G treated tongue Ep compared to IC animals at dpi1 (Fig. 7A). However, by dpi3 IS α-Ly6G animals with the most severe disease had significantly higher numbers of PMN-MDSCs compared to all other groups (Fig. 7A). Since MDSCs are known to have an immune-suppressive role in the context of tumor biology, we compared localization and proximity to Ca of PMN-MDSCs within IC α-Ly6G and IS α-Ly6G tissues that have equal CFUs but differ in Ca invasion depth (dpi3, Fig. 6E) by image deconvolution (Fig. 7B). PMN-MDSCs in IC α-Ly6G tissues showed peripheral positioning around Ca with little direct contact (Fig. 7B, upper panel). In contrast, IS α-Ly6G tissues showed dense foci of PMN-MDSCs (as shown by Arg1 + staining) that they were closely associated with Ca hyphae (Fig. 7B, lower panel). PMN-MDSCs from either group of α-Ly6G treated animals also showed expression of COX-2, suggesting that they might be undergoing autophagy^24^.

### Human PMN-MDSCs have little antifungal activity in vitro, but association with Ca hyphae increased their autophagy and survival.

Since PMN-MDSCs appear to be localized with Ca hyphae *in vivo*, we questioned whether they have any direct antifungal activity as do neutrophils. We compared Ca killing and phagocytic activity *in vitro* using freshly isolated human peripheral neutrophils (since they have higher activity than murine neutrophils) and human neutrophil-derived PMN-MDSCs. In contrast to previous reports ^23^, PMN-MDSCs had significantly lower (10-fold) phagocytic index (Fig. 7C) and had no fungicidal activity compared with that of neutrophils (Fig. 7D). Surprisingly, hyphal form Ca cells strongly attracted PMN-MDSCs (Fig. 7E) as we found in infected tissues *in vivo*. When co-incubated together, Ca yeast cells showed very little association with neutrophil-derived PMN-MDSCs *in vitro*, while Ca hyphae were found to be tightly associated with the surfaces of PMN-MDSCs (Fig. 7E). Since we observed COX-2 expression *in vivo*, this suggested that PMN-MDSCs-Ca hyphal association might induce autophagy in order to promote PMN-MDSC survival. Indeed, Ca hyphae increased PMN-MDSC autophagy by two-fold over yeast cells, and these levels were 70% of the positive control with rampamycin (Fig. 7F). Since autophagy inhibits MDSC apoptosis and promotes survival in the tumor microenvironment ^25^, we examined MDSC survival following exposure to Ca. PMN-MDSC survival was increased by 50% after 60–90 min exposure to Ca hyphae compared to unexposed cells (Fig. 7G), and was statistically equal to rampamycin treatment. In total these data show that PMN-MDSCs, although they have no direct antifungal activity, can strongly associate with Ca hyphae that promote their survival time.

## DISCUSSION

While neutrophils are essential first responders to clear fungal infection, our understanding of their role has been that pro-inflammatory signaling recruits an optimal quantity of neutrophils to the infection site. This work shows that a much more complex three-dimensional recruitment of neutrophils, but not macrophages, are needed for functional clearance of Ca in tissues as shown by the ability of cortisone to disrupt this morphology. We are the first to identify *in vivo* neutrophil swarms (previously described *in vitro* ^15, 26^) that are spatially associated with PMN-MDSCs within the oral mucosa. The mere presence of high numbers of neutrophils is not sufficient for *C. albicans* clearance, as observed following cortisone treatment in which elevated numbers of infiltrating neutrophils in the mucosa are unable to control infection. Although one previous work showed that MDSCs are found in peripheral blood following systemic infection by Ca ^23^, we are the first to discover that localized mucosal infection by Ca recruits PMN-MDSCs that are part of the neutrophilic swarm complex. Furthermore, we found no role for macrophages in the host response to oral candidiasis, unlike Ca infection in other tissues such as lung or kidney.

Neutrophil swarming is an emergent behavior triggered when neutrophils encounter targets that are significantly larger than individual neutrophils such as damaged tissues ^26^, clusters of bacteria ^27^, fungal hyphae ^28^, or parasites ^29^. During swarming, neutrophils communicate with each other, engaging in positive feedback signaling loops ^30^ driven exclusively by LTB4 in mice ^31^, and by LTB4 and other cytokines in human neutrophils ^26, 30^. Several features of the swarming process are truly unique and could not have been expected from previous knowledge of neutrophil fundamental activities e.g., chemotaxis, phagocytosis, enzyme and reactive oxygen release. These features, which were revealed in the past few years, include the transcellular synthesis of LTB4 during swarming ^31^, which acts as a relay between neutrophils ^32^, complement activation by neutrophils ^33^ and coordinated cytokine release ^30, 34^. Cytokines such as G-CSF and IL-1 are critical for *C. albicans* clearance as demonstrated by IL-1R KO studies ^7^. IL-17 has also been shown to contribute to *C. albicans* clearance during OPC, however, its role appears less relevant for neutrophil recruitment ^6^. We showed that *C. albicans* infection elicited a cytokine cascade involving IL-1 and G-CSF which resulted in the local production of neutrophil-specific chemokines such as CXCL-1 and CXCL-2. Disruption of this cytokine cascade by cortisone at an early stage of infection resulted in delayed and defective swarm formation, reduced PMN-MDSC recruitment and *C. albicans* overgrowth and biofilm formation.

Correlations have been also demonstrated between neutrophil swarming and both sustained ^35^ and transient ^36^ calcium fluxes in individual neutrophils, and the repetitive calcium waves centered on the target during the initiation of swarming ^37^. Moreover, factors limiting the size of the swarms have also been identified, including lipid mediators like lipoxin A4 ^30^ and resolvin D1 ^38^, intracellular signaling pathways that desensitize key receptors ^27^, and the activation of NADPH oxidase terminating the calcium waves ^37^. The swarming behavior of neutrophils is often perturbed in patients, in a broad range of conditions that associate with higher frequency of infections including cirrhosis, solid organ transplant, stem cell transplant, chronic granulomatous disease (CGD) ^13, 39, 40^. We observed that cortisone treatment downregulated the expression of BLT1 leading to defective swarm formation. However, it is possible that cortisone also impacts expression of lipoxins or resolvins resulting in sustained inflammation in the site of infection. Thus, restoration of components of the LTB4/BLT1 pathway could be a therapeutic intervention, as shown in a patient where restoring the swarming activity of neutrophils correlated with a reduced rate of infections ^14^.

We show for the first time that PMN-MDSCs are induced systemically in the bone marrow and recruited into the oral epithelium in response to localized *C. albicans* oral infection where they locate to the periphery of the neutrophil swarms. PMN-MDSCs were shown to have a protective role during murine systemic candidiasis by limiting host hyperinflammatory responses ^23^. However, this study found that adoptive transfer of neutrophilic MDSCs did not reduce kidney CFUs and had no protective effect in immunosuppressed mice ^23^. Our results suggest that Ca kidney infection (resulting from systemic candidiasis) may also require neutrophil swarming and localized PMN-MDSCs that may explain these findings.

The complete role of PMN-MDSCs in oral *C. albicans* clearance remains to be defined. Although we found anti-Ly6G treatment significantly depleted neutrophils (as previously shown in response to *C. albicans* infection ^41^), PMN-MDSCs were not affected and were found surrounding *C. albicans*. Despite such PMN-MDSC infiltration, *C. albicans* invaded beyond the basement membrane, and this invasion was significantly deeper (up to 200 µm) when neutrophil depletion was combined with cortisone. Thus, PMN-MDSCs might contribute immunosuppressive functions that permit deeper Ca invasion without exerting any direct fungicidal activity. However, *C. albicans* appears to communicate with PMN-MDSCs through co-localization and by inducing expression of COX-2, a marker of autophagy^24^. Thus, there is likely bi-directional signaling between PMN-MDSCs and neutrophil swarms as well as between PMN-MDSCs and *C. albicans* cells.

Thus, we find that *in vivo* neutrophil swarms containing PMN-MDSCs are essential for clearing oral *C. albicans* infection, and disruption of this function by cortisone is a mechanism for increased disease severity under immune deficient clinical settings. We show that effective host defense against *C. albicans* requires complex interactions among neutrophil swarms and PMN-MDSCs as key mechanisms of innate immunity.

## MATERIALS and METHODS

### Murine model of oral candidiasis.

Immunocompetent (IC) or Immunosuppressed (IS) C57BL/6J, 6–8 week-old female mice were infected sublingually as previously described ^9^. Animal protocols were approved by the University at Buffalo Institutional Animal Care and Use Committee (ORB06042Y). Each experimental group consisted of 5–8 mice, and experiments were repeated at least twice. Mice were immunosuppressed using 225 mg/kg of body weight cortisone 21-acetate (Sigma Aldrich C3 130-5G). Mice were anesthetized with a dose of Ketamine (100 mg/kg) and Xylazine (10 mg/kg) and infected sublingually with cotton balls carrying 1×10^7^*C. albicans* cells (CAI4-URA + with URA3 replaced at the RPS1 locus using a CLP10 plasmid or SC5314) for 1 h. Mice were monitored daily for weight loss. On days 1, 3 and 5 post-infection (dpi) mice were weighed, and sacrificed by cervical dislocation under anesthesia and tongues were harvested immediately. To quantify *C. albicans*, one-half of the tongue tissue was weighed, homogenized in phosphate buffer and plated on yeast extract-peptone dextrose (YPD) agar plates for 48 h to obtain the number of CFU/g of tissue. The other half of the tongue was fixed in 10% formalin and embedded in paraffin, to obtain 4µm sections that were processed for periodic acid Schiff (PAS) or Hematoxylin & Eosin stain for histological analysis and for immunohistochemistry. Whole tongues were obtained for epithelial and connective tissue dissection and protein extraction assays.

### Immunohistochemistry and histological analysis.

MPO+ (a neutrophil marker), CD163+ (a macrophage marker), Ly6G+ (a granulocytic cell marker), arginase+ (MDSC surrogate marker), and BLT1+ (leukotriene receptor marker) cells were detected by immunohistochemistry in 4µm tissue sections obtained from formalin-fixed, paraffin-embedded tongues using a Dako Omnis autostainer. Following antigen retrieval, slides were incubated for 20–30 min with primary antibodies against myeloperoxidase (1/200; Abcam # ab45977, Cambridge MA), CD163 (1/300; Abcam # ab182422), Ly6G (1/500; BD Pharmingen 551459), arginase 1 (1/1,200; Abcam #ab233548), BLT1 (1/200; Abcam, #ab188886), or COX-2 (1/100, Abcam # ab179800). Rabbit Envision (Agilent K4003) was applied for 30 min, followed by 5 min of incubation with 3,3′-diaminobenzidine (DAB, Dako) for visualization. Slides were counterstained with hematoxylin. H&E and immunostained slide sections were analyzed in at least ten low magnification digitalized counting fields for each epithelial and connective tissue compartment (5x magnification). Slide digitalization was obtained with either APerio scanner or with a Zeiss AxioScope A.1 microscope connected to a Zeiss Axiocam 105 color, and using ZEN 2011 Software. Number of cells / mm^2^ were obtained for Ly6G + MPO + granulocytes / neutrophils, CD163 + macrophages, and arginase + MDSCs in each tissue compartment (epithelium and connective tissue). Image deconvolution of PAS and Arg1 stained slides was performed at the Optical Imaging and Analysis facility of the University at Buffalo School of Dental Medicine. Research pathology services for this study were provided by the Pathology Network Shared Resource, which is funded by the National Cancer Institute (NCI P30CA16056) and is a Roswell Park Comprehensive Cancer Center Cancer Center Support Grant shared resource.

## Protein extraction, MPO ELISA and Multiplex ELISA assays

Freshly extracted whole tongues were treated with dispase at 37°C for 1 h to dissect the epithelium from the underlying connective tissue as previously described ^42^. Total protein was isolated from tongue epithelial or connective tissue using NP-40 buffer containing protease inhibitor cocktail (Thermo Scientific, Rockford, IL). Connective and epithelial tissues were homogenized in NP-40 buffer and sonicated. After centrifugation, total protein concentrations were determined using the BCA (bicinchoninic acid) protein assay (Thermo). Dissected tongue tissues were analyzed for the presence of MPO (Mouse myeloperoxidase DuoSet ELISA, R&D systems) and IL-1β, IL-1α, TNFα, G-CSF, CXCL1/ KC and CXCL2 / MIP-2, IL-17 (Bio-Plex^™^ Pro mouse custom assays, BioRad, Hercules CA, USA). The analysis was performed in duplicates on a Luminex 200 (Millipore), located at the Department of Flow and Image Cytometry, Roswell Park Cancer Institute.

## Collection of cells from murine blood, spleen and bone marrow

Blood was collected by heart puncture and collected in K2-EDTA tubes (BD) on the day of the assay under anesthesia, as described above. At least 200 µl of blood was obtained from each mouse. Femurs and spleens were harvested from mice after euthanasia by cervical dislocation under anesthesia. Bone marrow was flushed from femurs using fresh α-MEM media, passed through a 70 µm strainer using ice-cold HBSS without Ca+, pelleted by centrifugation, and resuspended in assay buffer. Spleens were homogenized using complete RPMI media and passed through a 70 µm filter. Erythrocytes were lysed with cell lysis buffer (BioLegend^®^, San Diego, CA).

## Flow cytometry

All antibodies were from BioLegend (San Diego, CA), unless stated otherwise. Blood (100 µl) and bone marrow cells (1 × 10^6^ in 100 µl of FCM buffer) (Leinco Technologies, St. Louis, MO) were first incubated with Fc block (TruStain FcX) and then stained with Live / Dead Aqua fluorescent reactive dye (Invitrogen), FITC conjugated anti-CD45 (30-F11), APC conjugated anti-CD11b (M1/70), PE conjugated anti-Ly6G (1A8), and PerCP/ Cy5.5 conjugated anti-Ly6C for 20 min at 30°C. Forward scatter versus side scatter was set to include singlets. By gating on CD11b^+^ cells, the numbers of neutrophilic (Ly6G^+^Ly6C^low^) and monocytic (Ly6G^−^Ly6C^hi^) cells were determined. Cells were run on a LSR Fortessa flow cytometer and data were analyzed by FlowJo (BD). Cytometry services were provided by the Flow and Image Cytometry Shared Resource at the Roswell Park Comprehensive Cancer Center, supported in part by the NCI Cancer Center Support Grant 5P30 CA016056.

## PMN-MDSC enrichment and T cell suppression assays

Suppression of T cell proliferation was used to functionally identify the expansion of PMN-MDSCs in spleen and bone marrows of mice infected orally with *C. albicans* as previously described ^43^. Briefly, single cell suspensions from spleen and bone marrows were enriched for Ly6G^+^ cells using a magnetic bead MDSC isolation kit according to the manufacturer’s instructions (Myltenyi Biotec, Ausburn, CA) and confirmed by flow cytometry. Splenocytes from uninfected C57BL/6J mice were used as naive T cell targets and stained with 5 µM CellTrace^™^ Violet (CTV). Spleen or bone marrow-derived Ly6G + enriched cells were then co-incubated in triplicate with CTV-stained splenocytes, at a ratio of 1:1 and 1:2 in 96-well round-bottomed plates coated with anti-CD3 (BD Pharmingen, San Jose, CA). After 72 h, cells were collected, labeled with PE conjugated anti-CD4 (clone RM4-5) and BV785 conjugated anti-CD8 (clone 53 – 6.7) mAb (BioLegend) and analyzed by flow cytometry. The percentage of suppression was calculated by (ΔMFI anti-CD3-stimulated T cells minus ΔMFI Ly6G^+^ cells-cocultured T cells) divided by ΔMFI anti-CD3-stimulated controls, where the difference in median fluorescence intensity (ΔMFI) was calculated by subtraction from unstimulated controls.

## Neutrophil depletion

For neutrophil depletion, mice were treated once with 150 µg per mouse of anti-Ly6G (clone 1A8, BioXcell) one day prior to infection, and daily with 10 µg per mouse of anti-G-CSF (clone 67604, R&D Systems), starting at day − 1 until dpi 3 as previously described ^7^. All injections were intraperitoneal (i.p.). Depletion of neutrophils was confirmed by flow cytometry by determining the number of CD45^+^CD11b^+^Ly6C^low^ cells.

### Production of human PMN-MDSCs.

Human PMN-MDSCs were induced as previously described ^24^ with some modifications. Human blood-derived neutrophils (H-PMNs) were isolated from venous blood purchased from Roswell Park Cancer as approved by the University at Buffalo Institutional Review Board (IRB; STUDY00006101). Blood was collected into VACUETTE EDTA tubes (Greiner Bio-One) from healthy donors, then cells were isolated by density gradient centrifugation using 1-step Polymorphs (Accurate Chemicals & Scientific Corporation). H-PMNs were cultured in T-25 flasks at 1X10^6^ cells/mL in complete medium, supplemented with IL-6 (20 ng/mL, MilliporeSigma) and GM-CSF (20 ng/mL, MilliporeSigma) for 7 days. H-PMNs cultured in medium alone were run in parallel as a control. Cultures were run in duplicate, and medium and cytokines were refreshed every 2–3 days. After one week, all cells were collected, and adherent cells were removed using the non–protease cell detachment solution Detaching (Genlantis). MDSC populations were characterized using CD14 and CD33 markers by flow cytometry. CD33 + cells were isolated from each culture using EasySep HLA Chimerism CD33 Whole Blood Positive Selection Kit (STEMCELL Technologies). The purity of isolated cell populations was determined to be greater than 90% by flow cytometry.

### Phagocytic Index and intracellular survival of C. albicans within human neutrophils or PMN-MDSCs.

Survival assays were performed as described previously ^44^. *C. albicans* cells and granulocytic cells (neutrophils or PMN-MDSCs) were incubated in RPMI 1640 medium for 3 h at 37°C and 5% CO_2_ at an MOI of 0.1. After 3 h of incubation, neutrophils were lysed with sterile water and 0.25% SDS (ThermoFisher) to release phagocytosed *C. albican.* Cell suspensions were collected, vortexed vigorously to avoid cell clumping, and were plated on YPD BD Difco agar and incubated for 48 h at 30°C to obtain viable CFU. Cells incubated in RPMI 1640 medium were used as control. Percent survival was calculated using the following formula: (*C. albicans* CFU in control medium - CFU in neutrophils or PMN-MDSCs cells medium/ CFU in control medium) X 100. In order to obtain the number of *C. albicans* internalized by granulocytes, phagocytic index assays were performed in parallel. Human neutrophils or PMN-MDSCs and *C. albicans* were co-incubated as indicated above, then cell suspensions were collected and placed in positively charged slides (Globe Scientific Inc.) to allow cells to adhere. Following adhesion, supernatants were gently removed and non-phagocytosed *C. albicans* were stained with 4 mg/mL of Calcofluor White (Sigma-Aldrich) for 2 min, on ice. Cells were washed with ice-cold PBS 1X and fixed with 4% paraformaldehyde (Electromicroscopy Science) for 30 min at 21°C. Cells were permeabilized with 0.1% Triton^™^ X-100 (Fisher Bioreagents^™^) for 5 min, then stained with 4 mg/mL Alexa-Fluor 488 phalloidin (Invitrogen). Cells were mounted with #1 cover glass (Knittel Glaeser) and fluorescent mounting medium (Dako) and internalized *C. albicans* cells were visualized using Zeiss Axio Observer Z1 inverted fluorescent microscope (Carl Zeiss, Germany). The phagocytic index was calculated as (total number of internalized yeast / total neutrophils or PMN-MDSCs counted). Survival and phagocytic index assays were performed in triplicates and results are representative of at least three independent experiments.

## Statistics

Data were analyzed using Tukey-Kramer, Mann-Whitney, or two-tailed Student’s t-tests, according to data distribution, and GRAPHPAD PRISM software V 7.0 (Graph Pad Software Inc. CA, USA). *P* values < 0.05 were considered statistically significant.
